# The fairness paradox in public regulatory summaries of FDA-authorized artificial intelligence-enabled medical devices

**DOI:** 10.3389/fmed.2026.1894905

**Published:** 2026-08-17

**Authors:** Chun-Wang Lui, Pusheng Wang

**Affiliations:** 1 Tsinghua Shenzhen International Graduate School, Tsinghua University, Shenzhen, China; 2 Technological and Higher Education Institute of Hong Kong, Hong Kong SAR, China

**Keywords:** algorithmic accountability, contestable fairness, FDA-authorized AI-enabled medical devices, performative transparency, sociotechnical systems

## Abstract

The U. S. Food and Drug Administration has published several types of public regulatory summaries of artificial intelligence-enabled medical devices, including 510(k) summaries, *De Novo* decision summaries, and premarket approval summaries of safety and effectiveness data. These documents are among the few regulatory texts routinely accessible to clinical institutions, patient advocates, educators, and researchers. Existing empirical audits have documented reporting deficits in areas such as demographic representation, model development details, and clinical validation data. This Perspective draws on these empirical findings and sociotechnical scholarship to formulate the saturation-gap paradox as a preliminary conceptual hypothesis requiring future empirical validation. It proposes that public summaries may be technically and procedurally detailed while providing only limited entry points for assessing fairness, clinical equity, and accountability. Rather than presenting a new systematic audit or inferring what FDA reviewers evaluated internally, this perspective focuses on the accessibility, structure, and accountability function of public disclosures. It considers three ways in which these disclosure genres may limit fairness scrutiny. These involve semantic abstraction, metricization and procedural governance, and accountability redistribution. Finally, building on this prioritized conceptual framework, this Perspective offers preliminary design propositions for disclosure reform and an illustrative TPACK-based educational outline as directions for future research, stakeholder consultation, and prospective development.

## 1 Introduction

Artificial intelligence-enabled medical devices are rapidly entering clinical practice under the U. S. Food and Drug Administration (FDA) regulatory framework (1), with many products reaching the market through the 510(k) pathway and others through the *De Novo* or premarket approval (PMA) pathways (2). These publicly available summaries may inform institutional procurement, clinical implementation, professional education, and public understanding of regulatory decisions. The practical value of disclosure depends on whether stakeholders can use the available information to assess fairness, clinical equity, and the allocation of responsibility. Even when public summaries report subgroup performance or risk-management measures, they may not clearly explain how benefits and burdens are distributed, who is responsible for addressing harms, or how affected stakeholders can question decisions and seek remedy.

When summaries foreground technical performance metrics and procedural language, rights-based concerns, distributive justice, and clear lines of accountability may receive less attention, even when subgroup performance or risk management is reported (3, 4). This communicative limitation is directly relevant to healthcare professions education because clinicians increasingly need to read regulatory texts as part of responsible AI adoption.

Many public summaries emphasize subgroup performance, sensitivity, specificity, risk management, and post-market surveillance, but these technical categories do not always translate into a clinically interpretable account of fairness, equity, or contestability (5). Subgroup analyses of sensitivity, specificity, calibration, and error rates remain essential for identifying disparities. Their value for fairness assessment depends on whether the results are interpreted in relation to clinical consequences, normative judgments, responsibility allocation, and available channels for remedy.

Technically detailed public disclosures may appear comprehensive while leaving some fairness-relevant questions difficult to articulate in the public record (3, 6). The issue is not simply how much information is available, but whether stakeholders can use it to question fairness claims, identify responsibility, and seek review or remedy (7). We introduce the saturation-gap paradox as an original conceptual hypothesis to characterize this tension. The concept draws on published audits of FDA-authorized AI-enabled medical device summaries (5, 8) and sociotechnical scholarship on transparency and abstraction (3, 9). We explicitly frame the saturation-gap paradox as a conceptual hypothesis rather than an established empirical finding. It serves as an interpretive heuristic intended to guide future empirical coding and quantitative audits of FDA summary texts. We do not claim that technical language directly excludes fairness concerns, and whether the proposed pattern shapes clinical or public understanding remains an empirical question.

Clarifying this tension requires distinguishing among several related concepts. Demographic representation describes whether development and evaluation data reflect the intended patient population, while subgroup performance captures measured differences across clinically relevant groups. Generalizability refers to the extent to which performance observed in evaluation data is likely to hold across different populations, sites, workflows, equipment, and operating conditions [FDA et al., 2021 (10)]. Statistical fairness refers to formal quantitative criteria for comparing model behavior, whereas clinical equity addresses avoidable differences in access, safety, workload, clinical consequences, or outcomes. Nondiscrimination focuses on unjustified differential treatment, while distributive justice examines how benefits, burdens, and risks are allocated (9, 11, 12). Contestability describes the capacity of affected stakeholders to question an AI-supported decision or governance arrangement, introduce contextual evidence, obtain review, and seek correction or remedy (7, 13). Further operational definitions, measurable indicators, and supporting sources are provided in Supplementary material 1.

We focus on publicly available FDA documents associated with the 510(k), *De Novo*, and premarket approval pathways for AI-enabled medical devices. FDA-authorized AI-enabled medical devices may be cleared through 510(k), granted marketing authorization through the *De Novo* pathway, or approved through PMA. These pathways produce different public documents and involve different evidentiary expectations. A 510(k) summary typically describes the device, performance testing, and basis for substantial equivalence. A *De Novo* decision summary explains the classification and evidence supporting authorization of a novel low- or moderate-risk device. For PMA approvals, FDA prepares a detailed Summary of Safety and Effectiveness Data (SSED) and makes it publicly available. The SSED describes the device, relevant nonclinical and clinical evidence, and FDA’s assessment of safety and effectiveness (26). Given this broader evidentiary scope, the discussion centers on 510(k) and *De Novo* summaries, while SSEDs serve as a secondary point of reference.

Information submitted by manufacturers, information evaluated by FDA reviewers, and information disclosed publicly are not equivalent. The absence of explicit fairness or subgroup information from a public summary does not establish that these issues were not considered during regulatory review. Our critique is limited to the accessibility and structure of public disclosures and their capacity to support accountability. Assessing confidential submissions or internal FDA review processes would require separate evidence.

**Figure 1:**
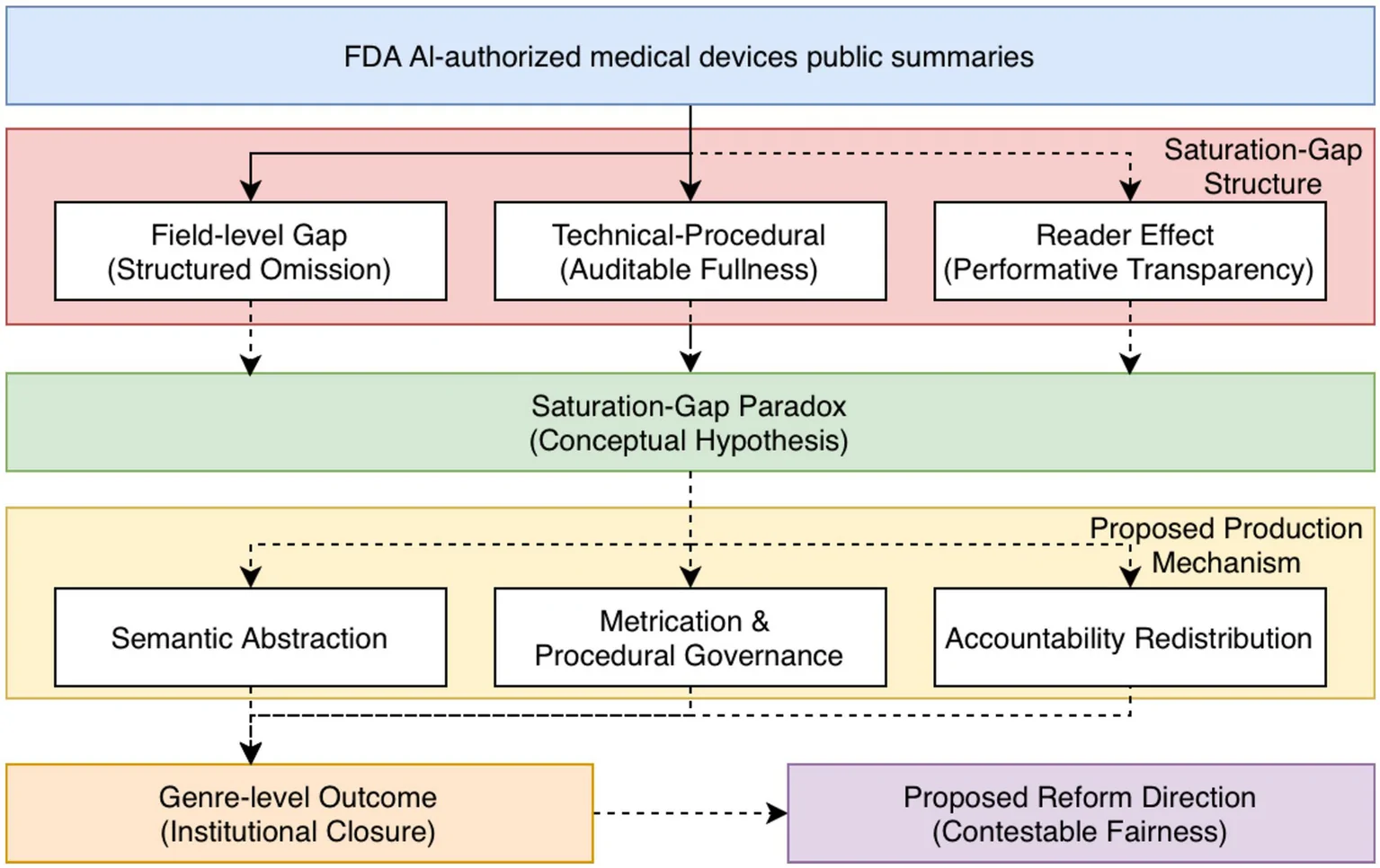
Conceptual framework of the saturation-gap paradox and prospective reform directions. Solid arrows represent empirical relationships supported by published reporting audit literature. Dashed arrows represent proposed theoretical mechanisms, interpretive reader effects, and conceptual hypotheses requiring future empirical validation.

Within this scope, we consider three ways in which the structure of public summaries may limit fairness scrutiny. Semantic abstraction may translate clinical and social concerns into simplified technical categories (9). Metricization and procedural governance may emphasize aggregate performance measures over local clinical context (4). Accountability redistribution concerns the possibility that healthcare institutions and frontline clinicians may be left to manage unresolved uncertainty and monitoring responsibilities (11). We treat these pathways as interpretive propositions rather than established causal mechanisms. Figure 1 summarizes their proposed relationships. While this Perspective prioritizes the conceptualization and theoretical mechanisms of the saturation-gap paradox, it concludes by outlining a preliminary disclosure framework and an exploratory TPACK application as prospective future directions to guide subsequent empirical validation and pedagogical development. Future document-level coding and qualitative case studies could test, refine, or challenge the framework. Methodologically, this Perspective is informed by a targeted review of official FDA regulatory policy documents and lifecycle guidance frameworks (including PMA application content specifications, Predetermined Change Control Plan (PCCP) guidance, Good Machine Learning Practice (GMLP), and Transparency Guiding Principles). Literature evidence was selected through keyword searches focusing on FDA-authorized AI medical devices, empirical reporting audits, and algorithmic fairness policy scholarship, which were synthesized to construct the proposed conceptual framework.

## 2 Saturation and gaps

Existing audits document reporting omissions in public summaries, including deficits in baseline model characteristics (14), clinical benefit–risk and demographic data (15), localized reporting gaps (5), and geographic validation blind spots (10). These studies provide the empirical starting point for, but do not by themselves prove, the saturation-gap paradox. Representative examples from this literature show a recurring mismatch. Public summaries may report aggregate performance metrics, device specifications, validation procedures, and risk-management language, while reporting demographic composition, subgroup-specific clinical consequences, external validation settings, and post-market accountability mechanisms less consistently. The saturation-gap paradox names this mismatch between technical disclosure density and the more limited availability of publicly contestable fairness information. It should be read as a conceptual explanation to be tested, not as evidence that technical density necessarily determines the absence of fairness-oriented disclosure.

Examining the political implications of regulatory transparency requires shifting focus from traditional inquiries about missing fields to analyzing what the core narrative foregrounds and whom the institutional rules empower to initiate normative critiques. Recent evaluations suggest that even when internal algorithmic characteristics are disclosed, they may remain framed within administrative technical parameters (8). This aligns with observations by regulatory agency authors that FDA communications provide safety and effectiveness information, although the level and presentation of detail may not meet the needs of all stakeholders (16). This divergence between surface-level visibility and structural accountability indicates that merely expanding disclosure volume may not by itself overcome existing institutional filters. Without altering the internal textual allocation and stylistic grammar of public regulatory genres, newly mandated disclosure fields may be incorporated into the dominant technical-administrative genre without creating meaningful space for fairness contestation (17). Crucially, this critique aims to delineate the technocratic boundaries of current disclosure reforms rather than dismissing them entirely. If interventions stop at completing checklists or filling templates, fairness may remain procedurally narrowed by technical syntax. Regulatory critique consequently requires a shift from a singular pursuit of informational volume to the preservation of contestability, evaluating whether public texts afford legitimate entry points for normative accountability.

## 3 Three mechanisms

We organize the argument around three mechanisms that may influence how fairness concerns are presented in public regulatory summaries. These mechanisms are interpretive propositions and do not establish regulatory intent or replace document-level analysis.

The three mechanisms are presented as an interconnected, potentially self-reinforcing process rather than as independent categories. First, semantic abstraction may translate open-ended normative equity concerns into simplified machine-learning attributes (9). Second, metricization and procedural governance may stabilize the public narrative by foregrounding performance metrics and procedural language, including AUC, sensitivity, specificity, verification, validation, and risk management. These categories are necessary for safety and effectiveness evaluation, but when presented without clinical interpretation or accountability pathways, they can make alternative ethical questions harder to raise within the public text. Third, limited public contestability can contribute to accountability redistribution, whereby residual uncertainties about local performance, workflow integration, or post-market drift are managed primarily by downstream clinical environments. This framework does not assume that regulators or manufacturers deliberately transfer responsibility. Public documents may nevertheless leave unclear who is expected to monitor, report, interpret, and respond to fairness-related concerns after authorization.

### 3.1 Semantic abstraction: the technical translation of fairness issues

The primary pathway involves translating substantive distributive conflicts into technically abstract terminology. Within public texts, normative debates concerning structural bias and rights claims may be reframed through statistical and machine learning concepts, including subgroups, generalizability, statistical variation, and representation. A critical reading of these disclosures suggests that the term representation may refer to data structures or sample characteristics rather than demographic or social representation (9, 16). This form of abstraction may make the structural sources and clinical consequences of inequity less visible.

Technical categories are essential for assessing subgroup performance and generalizability. However, they provide only a partial account of fairness unless observed differences are interpreted in relation to social context, clinical consequences, and institutional responsibilities (FDA et al., 2024). FDA guidance on predetermined change control plans (PCCPs) further recommends a description of modifications, modification protocol, and impact assessment, together with public-facing information about planned modifications, testing, validation, performance requirements, and communication to users (27).

### 3.2 Metricization and procedural governance: the substitution of normative disputes

The secondary mechanism describes how normative debates on social justice may be displaced by quantitative metrics and administrative procedural syntax. In public summaries, fairness-relevant questions may be framed primarily through technical performance metrics and compliance language, including area under the curve (AUC), sensitivity, specificity, verification, validation, risk management, and post-market surveillance. These tools are necessary for safety and effectiveness evaluation. However, when they are not connected to clinical consequences, power asymmetries, or pathways to remedy, they may narrow the evaluative space for addressing systemic harm. Under this narrative regime, explainability can remain limited to technical descriptions of how an algorithm computes an output, rather than supporting contestability, which requires justifying whether a decision is acceptable under broader social and ethical norms (6, 11, 18).

To fully operationalize this framework, it is crucial to delineate two structural dimensions highlighted in our conceptual model in Figure 1. First, the field-level gap refers to the limited presence of dedicated, formalized evaluation blocks within regulatory summary templates, which may restrict the generation of equity-oriented narratives.

Second, performative transparency may influence how readers interpret regulatory summaries. Dense compliance information may create an appearance of comprehensiveness while leaving substantive equity questions difficult to identify (3). This is proposed as an interpretive possibility rather than an empirically established cognitive response among clinicians. Its relevance should be evaluated in future studies of how clinicians, procurement committees, patients, and educators interpret public regulatory summaries.

### 3.3 Accountability redistribution: the clinical spillover and assumption of technical risks

The tertiary mechanism operates through the textual reconfiguration of algorithmic accountability, where latent technical uncertainties may be shifted toward clinical front lines. Public summaries often center on authorization pathways and technical specifications, potentially leaving clinicians and healthcare institutions to interpret how residual uncertainty should be managed in local workflows (11). Post-market validation gaps and early recalls further illustrate why downstream environments may need clearer information about monitoring and escalation (1). We propose accountability redistribution as a conceptual description of this communicative pattern rather than evidence of deliberate risk transfer by any single actor. Current public summaries may verify conformity with auditable requirements while providing less explicit guidance on responsibility for distributive fairness and locally observed inequities (17).

## 4 Current FDA lifecycle governance and remaining disclosure gaps

FDA’s evolving total product lifecycle approach provides important context for the proposed framework. The good machine learning practice (GMLP) guiding principles emphasize multidisciplinary expertise, representative development and test datasets, subgroup performance, clinically relevant testing, clear user information, and monitoring of deployed models for performance degradation, retraining-related risks, and unintended bias (28). The subsequent transparency for machine learning-enabled medical devices guiding principles adopt a human-centered approach organized around who receives information, why it is needed, what is communicated, where and when it is provided, and how communication is supported throughout the lifecycle (29).

These initiatives substantially strengthen lifecycle governance, but submission, regulatory review, public disclosure, post-authorization updating, and clinical use involve different information needs. Manufacturers may provide detailed development, validation, risk-management, and change-control information in their submissions, which FDA evaluates against the applicable authorization standard. Only a subset of this material appears in public summaries and labeling because of legal and confidentiality constraints. Following authorization, labeling, version information, PCCP-related modifications, and monitoring information may need to be updated. Clinicians and patients, meanwhile, need concise information that supports interpretation, local implementation, reporting, and response. The remaining question is whether the information made public across these stages is sufficiently clear to assess demographic representativeness, subgroup consequences, performance drift, responsibility allocation, and routes for contesting suspected inequities.

## 5 Prospective directions

Pursuing additional disclosure fields without redesigning their accountability function risks keeping regulatory reforms within the confines of checkbox transparency. While the number of reported indicators may expand, substantive fairness disputes may still lack institutionalized public entry points (19). Therefore, we propose shifting attention from the volume of information disclosed to whether disclosures support auditability and public scrutiny. This proposal aligns with principles of algorithmic contestability, which emphasize opportunities for human challenge, alternative interpretation, and localized normative dispute (7, 13, 20). Applying contestable AI by design to administrative workflows would require public documents to provide meaningful channels for inquiry rather than only additional technical fields (6). These proposed disclosure components are presented not as definitive policy mandates, but as a preliminary prospective framework for future empirical evaluation.

Enabling fairness to be meaningfully contested rather than simply measured may require restructuring the textual grammar of future public summaries. Prior literature has established general frameworks for integrating contestability into algorithmic system design (7, 20). We adapt these principles to public medical device disclosures through dedicated sections on fairness harms, unassessed populations, responsibility allocation, and routes to remedy. Their implementation would require coordination across the medical device ecosystem. Regulators could consider normative review channels alongside technical assessment, while sponsors could articulate equity-relevant claims and post-market responsibilities more explicitly (21, 22). These proposals aim to make public regulatory summaries more useful for scrutiny and accountability. They are designed to complement existing approaches to safety and effectiveness evaluation.

To illustrate how public summaries might be re-oriented to support contestability rather than volumetric disclosure alone, we outline four preliminary design propositions. These components are presented as illustrative applications and directions for future research and policy exploration, rather than complete, validated, or implementation-ready regulatory standards. First, a demographics and omission narrative identifies represented, underrepresented, and unassessed populations, sites, devices, and data sources. It also explains clinically relevant omissions and resulting limitations. Manufacturers prepare the narrative for FDA assessment, and the information appears in or is linked from the public summary and current labeling. The narrative is updated when the intended population, device, or supporting evidence changes in a material way. Confidential commercial information can remain protected through aggregation, de-identification, ranges, and the omission of proprietary details, provided that the information needed for safe clinical interpretation is retained.

Second, an error-gap metrics module reports clinically relevant subgroup sensitivity, specificity, calibration, false-positive and false-negative rates, uncertainty intervals, acceptance criteria, and their clinical implications. The manufacturer generates the module and verifies it within its quality system. FDA reviews the information when it bears on authorization or a subsequent modification, and the module appears in the public summary or linked labeling. Updates follow performance-relevant modifications or material subgroup findings after authorization. Privacy can be protected through small-cell suppression, aggregation, and uncertainty reporting, so that unstable estimates are not treated as definitive findings.

Third, a responsibility allocation statement defines duties across manufacturers, healthcare institutions, clinicians, regulators, system operators, and relevant vendors. It covers updates, training, local validation, workflow integration, monitoring, reporting, investigation, patient communication, and escalation. Manufacturers prepare a baseline statement for regulatory assessment and public disclosure. Local institutions may add deployment-specific responsibilities without changing manufacturer obligations. The statement appears in the public summary or linked labeling and in institutional documentation. It is revised when changes to the device, workflow, or risk profile reallocate responsibility.

Finally, a contestability interface provides a manufacturer-maintained reporting route linked from public summaries and labeling. Institutional and regulatory pathways complement this route. The interface specifies who may report, what information is required, who triages concerns, and how follow-up is provided. Expected response times and escalation routes are also made clear. Reports of performance drift, subgroup error patterns, usability problems, or inequitable consequences may lead to investigation, risk mitigation, or corrective action. They may also prompt labeling or training updates, PCCP-related action, post-market surveillance, or communication with FDA. Patient information is minimized and protected. Public updates may report aggregate trends and resulting actions without disclosing individual reports.

This Perspective is conceptual in nature and does not present a document-level empirical analysis of FDA summaries. The saturation-gap paradox and its underlying mechanisms are proposed as an interpretive framework and conceptual hypothesis rather than demonstrated causal laws. Similarly, the four disclosure components outlined above serve as preliminary design propositions and directions for future research rather than a complete or implementation-ready framework. Operationalizing these proposals will require independent framework development, multi-stakeholder consultation (involving regulators, device manufacturers, and clinicians), pilot testing, and rigorous evaluation to assess their practical feasibility.

## 6 Exploratory applications

Lifecycle policy alone may not ensure that clinicians can interpret public disclosures or respond to their limitations. To address this educational gap, we draw on the technological pedagogical content knowledge (TPACK) framework. TPACK extends Shulman’s concept of pedagogical content knowledge by integrating technological, pedagogical, and content knowledge (23–25). In this application, public regulatory summaries provide the content base, knowledge of subgroup performance and lifecycle governance represents the technological component, and guided case analysis provides the pedagogical approach. This framework offers an establishes basis for embedding regulatory literacy within healthcare professions education. To demonstrate how regulatory critique might be integrated into health professions education, we outline an illustrative application based on the TPACK framework. This outline serves as an exploratory design proposition and an example of potential future educational practice, rather than an operational, off-the-shelf curriculum.

This educational activity could be used with senior medical students and residents in the early stages of postgraduate training and adapted for learners in nursing, pharmacy, and allied health professions. It aims to develop learners’ ability to distinguish among public documents associated with the 510(k), *De Novo*, and PMA pathways and to recognize limitations in demographic representation, validation, and subgroup reporting. Learners would also examine the clinical implications of subgroup performance, consider how responsibility is distributed among manufacturers, healthcare institutions, clinicians, regulators, and system operators, and identify suitable reporting or escalation routes when safety or equity concerns arise. Together, these skills support regulatory literacy, evidence interpretation, and responsible clinical engagement with AI-enabled medical devices.

A structured case-based session could use a current 510(k) summary, *De Novo* decision summary, SSED, or linked labeling as the primary text. Learners would first identify the authorization pathway and intended use, then extract population and subgroup evidence, compare the authorized population with a local clinical scenario, identify uncertainty or omission, and apply the proposed responsibility allocation and contestability modules. The session could combine a facilitated review of the document with small-group discussion and a short escalation simulation. Learners would consider how regulatory information informs procurement, workflow design, patient communication, and decisions about when to question or override an AI-supported output.

Assessment could take the form of a written case analysis, a short reflective brief, or a structured simulation. Across these formats, a shared rubric would focus on learners’ ability to identify the authorization pathway, interpret subgroup evidence, recognize clinically important uncertainty, explain how responsibilities are distributed, and choose a proportionate escalation response.

## 7 Conclusion

In summary, the saturation-gap paradox hypothesizes that how technically dense public disclosures may still leave fairness concerns difficult to challenge and accountability difficult to pursue. Public disclosure without meaningful opportunities for contestation may provide only limited support for accountability. Healthcare professions education should move beyond traditional biostatistics alone to include critical AI literacy and sociotechnical reading of regulatory summaries. Future clinicians should learn how to interpret performance metrics, identify unassessed demographic subgroups, trace responsibility allocation, and use reporting channels to challenge unsafe or inequitable deployments. By integrating regulatory critique into professional training, medical education can help clinicians become active stewards of clinical equity rather than passive adopters of AI-enabled tools.

Future empirical work should test whether revised disclosure templates can improve clinical comprehension, public scrutiny, and post-market accountability. The policy question is not simply whether more fields are added to public summaries, but whether those fields enable stakeholders to determine if enhanced transparency produces more substantive and contestable forms of fairness. The current FDA landscape illustrates both the promise and the limits of public transparency. Lifecycle initiatives such as GMLP and PCCPs strengthen governance, but public summaries should also support clinical learning, accountability, and fair implementation. The disclosure components and educational outlines discussed herein should be viewed as initial design propositions that invite empirical piloting and stakeholder engagement, rather than definitive mandates.

## Data Availability

The original contributions presented in the study are included in the article/Supplementary material, further inquiries can be directed to the corresponding author/s.
