# Supplementary material for: The fairness paradox in public regulatory summaries of FDA-authorized artificial intelligence-enabled medical devices
Source: Front Med (Lausanne). 2026 Aug 17;13:1894905. doi: 10.3389/fmed.2026.1894905 (PMC13526585; doi:10.3389/fmed.2026.1894905)
Supplement: Supplementary file 1 [file Data_Sheet_1.docx]

**Supplementary Material 1.**

This glossary defines the key terms as they are used in this perspective. The saturation-gap paradox is introduced as the authors’ original conceptual hypothesis. The three mechanisms and four disclosure modules are literature-informed constructs developed in this perspective. Their underlying concepts draw on prior scholarship, while their specific organization and application to U.S. Food and Drug Administration (FDA) public summaries are proposed here.

Table S1. Operational Codebook and Key Terms Glossary

| **Term** | **Construct Status** | **Operational definition in this perspective** | **Measurable Indicators & Operational Criteria of Core Constructs** | **Selected supporting sources** |
| --- | --- | --- | --- | --- |
| Public regulatory summary | Empirical Institutional Artifact | A publicly accessible document made available through FDA that communicates part of the basis for an authorization decision, including a 510(k) summary, De Novo decision summary, or PMA Summary of Safety and Effectiveness Data (SSED). It contains only part of the information submitted by the manufacturer and considered by FDA during regulatory review. | Document classification: Specific regulatory pathway (510(k), De Novo, PMA) and overall page/section coverage. | (FDA, 2025; FDA et al., 2021, 2024) |
| Saturation-gap paradox | Author-Proposed Conceptual Hypothesis | The authors’ original conceptual hypothesis that a public summary may be dense with technical and procedural information while still offering limited entry points for evaluating fairness, clinical equity, responsibility, and remedy. It is a heuristic to be empirically tested, not a demonstrated causal law and requiring future validation. | Co-occurrence Threshold: A document exhibits the paradox when it simultaneously satisfies ≥1 Technical Saturation Criterion AND ≥1 Fairness Gap Criterion defined below. | (Ananny & Crawford, 2018; Muralidharan et al., 2024; Selbst et al., 2019; Wu et al., 2021) |
| Technical saturation | Proposed Operational Heuristic (Requires future empirical testing) | The dense concentration of quantitative performance parameters and compliance details within public summary texts | Identified by meeting ANY of the following quantitative thresholds:  1. Metrics count: Inclusion of ≥5 distinct quantitative performance metrics (e.g., AUC, sensitivity, specificity, PPV, NPV, ROC);  2. Compliance terminology: Presence of ≥3 standardized regulatory/procedural terms (e.g., 510(k), GMLP, PCCP, ISO/IEC standards);  3. Detailed specifications: Inclusion of explicit model architecture, hardware specifications, or algorithmic parameters;  4. Validation protocol: Extensive technical descriptions of data splitting, cross-validation, or bench testing;  5. Technical proportion: ≥50% of total document word count devoted purely to engineering and statistical specifications. | (Mehta et al., 2025; Shick et al., 2024) |
| Fairness gaps | Proposed Structural Heuristic  (Requires future empirical testing) | The structural absence or incomplete reporting of demographic subgroup performance and clinical equity evaluation. | Identified by the presence of ANY of the following explicit criteria:  1. Subgroup data omission: Absence of subgroup-specific performance metrics (e.g., stratified ROC/AUC);  2. Demographic absence: Absence of demographic breakdowns (race, ethnicity, age, sex) for validation cohorts;  3. Unexplained omissions: No explanation provided for missing or underrepresented demographic groups;  4. Differential impact: No discussion of potential differential clinical risks or error patterns across patient populations;  5. Unassigned duties: Absence of an explicit post-market responsibility allocation statement;  6. Lack of contestability: No operational mechanism or channel for stakeholder challenge, correction, or appeal. | (Muralidharan et al., 2024; Wu et al., 2021) |
| Demographic representation | Established Regulatory Metric | The extent to which model development and validation cohorts reflect intended clinical patient populations. | Demographic reporting rate: Inclusion of stratified sample counts across relevant characteristics such as age, sex, race, ethnicity, disease severity, care setting, and measurement conditions. | (FDA et al., 2021; Shick et al., 2024) |
| Subgroup performance and generalizability | Established Technical Metric | Subgroup performance captures measured error rates across patient groups, while generalizability concerns cross-site/workflow transportability. | Disaggregated error evaluation: Subgroup-specific calibration curves, sensitivity/specificity breakdowns, and multi-site validation data. | (FDA et al., 2021; Muralidharan et al., 2024; Windecker et al., 2025) |
| Statistical fairness, clinical equity, nondiscrimination, and distributive justice | Established Interdisciplinary Concepts | Statistical fairness concerns formal quantitative criteria for comparing model behavior. Clinical equity concerns avoidable differences in access, consequences, workload, safety, or outcomes. In this Perspective, nondiscrimination concerns unjustified differential treatment, while distributive justice concerns how benefits, burdens, and risks are allocated. These normative questions cannot be resolved by a single metric. | Socio-clinical mapping: Contextualized evaluation connecting quantitative error disparities to real-world clinical harms and resource allocation. | (Binns, 2022; FDA et al., 2024; Friedler et al., 2016; Selbst et al., 2019) |
| Explainability | Established Technical Concept | Concerns whether the basis, operation, or outcome of a system can be understood (i.e., information transparency regarding model logic, feature importance, statistical parameters, and performance metrics). | Informational Metrics: Inclusion of explicit performance metrics (AUC, sensitivity, specificity, PPV, NPV), ROC curves, feature attribution descriptions, and model architecture specs | (Raji et al., 2020; Shick et al., 2024) |
| Contestability / contestable fairness | Established Governance Concept | Concerns whether affected stakeholders can question, challenge, appeal, correct, or seek accountability for a decision or omission. | Actionable Entry Points: 1. Inclusion of designated user dispute/reporting protocols; 2. Clear textual allocation of post-authorization responsibilities; 3. Explicit escalation channels for performance drift or subgroup harm | (Alfrink et al., 2023; Lyons et al., 2021; Mulligan et al., 2018) |
| Performative transparency | Proposed Interpretive Possibility  (Literature-informed hypothesis requiring future validation) | A literature-informed reader-facing condition in which dense or formal disclosure may create an appearance of comprehensiveness without necessarily enabling substantive scrutiny or accountability. In this manuscript it is an interpretive possibility requiring empirical validation. | Boilerplate Ratio: High proportion of generic regulatory template language relative to actionable local workflow guidance. | (Ananny & Crawford, 2018) |
| Semantic abstraction | Literature-Adapted Theoretical Construct | The translation of contextual, institutional, or normative concerns into simplified technical categories that may omit relationships, histories, and local clinical consequences. | Operational Matrix Evaluation (see Table S2 below): Document contains technical terminology that systematically substitutes substantive equity/distributive issues with procedural or statistical vocabulary. | (Selbst et al., 2019) |
| Metricization and procedural governance | Literature-Informed Interpretive Mechanism | The organization of evaluation around quantitative indicators, validation procedures, and compliance syntax. These tools are necessary for safety evaluation but may narrow inquiry when disconnected from clinical consequences and accountability. | Procedural focus ratio: Percentage of text devoted to compliance and verification protocols versus clinical impact analysis. | (Raji et al., 2022) |
| Accountability redistribution | Literature-Informed Interpretive Mechanism | A literature-informed description of how residual uncertainty and monitoring responsibilities may become operational burdens for downstream clinical environments when public disclosures do not clearly allocate responsibility. It does not imply deliberate risk transfer by a regulator or manufacturer. | Duty Allocation Text: Explicit textual assignment of drift detection, local validation, and clinical oversight to health systems vs. sponsors. | (Binns, 2022; Raji et al., 2020) |
| Demographics and omission narrative | Literature-Informed Proposed Module | A disclosure module that identifies represented, under-represented, and unassessed populations and explains clinically relevant omissions, limitations, update triggers, and confidentiality safeguards. | Structural coverage: Presence of a dedicated narrative section explicitly describing excluded patient groups and clinical limits. | (FDA et al., 2021; Shick et al., 2024) |
| Error-gap metrics module | Literature-Informed Proposed Module | A disclosure module that connects subgroup-specific error and calibration measures to clinical consequences, acceptance criteria, uncertainty, update triggers, and post-market evidence. | Metric integration: Presence of standardized error-gap reporting linked with clinical acceptance criteria and uncertainty ranges. | (FDA, 2025; Raji et al., 2020) |
| Responsibility allocation statement | Literature-Informed Proposed Module | A disclosure module that identifies responsibilities for updates, training, local validation, workflow integration, monitoring, reporting, investigation, communication, and escalation across manufacturers, institutions, clinicians, regulators, operators, and vendors. | Role clarity scores: Clear categorization of post-authorization monitoring, retraining, and clinical governance roles. | (Binns, 2022; Raji et al., 2020) |
| Contestability interface | Literature-Adapted Reform Module | A reporting and follow-up mechanism through which clinicians, patients, and institutions can raise concerns, receive triage and review, and connect evidence of drift, subgroup errors, usability problems, or inequitable consequences to appropriate action. | Feedback linkage: Inclusion of structured escalation channels, triage processes, and feedback loops within summary documents. | (Alfrink et al., 2023; Windecker et al., 2025) |
| FDA lifecycle frameworks | Existing Policy Artifact | An umbrella term used here for Good Machine Learning Practice (GMLP), the transparency guiding principles, and predetermined change control plan (PCCP) guidance. Together they address lifecycle development and monitoring, human-centered communication, planned modifications, validation, public-facing information, and communication to users; they do not eliminate pathway-specific legal requirements. | Lifecycle alignment: Reference rate and compliance mapping to official GMLP, transparency, or PCCP regulatory guidance. | (FDA, 2025; FDA et al., 2021, 2024) |

**Table S2. Operational Matrix for Semantic Abstraction Analysis**

| 1. Underlying Substantive Equity Issue | 2. Terminology Used in Regulatory Text | 3. Mechanism of Terminology Obscures / Depoliticizes | 4. Transparent Communication Alternative |
| --- | --- | --- | --- |
| Subgroup Disparity & Harm | A description of situations in which the device may fail or may not operate at its expected performance level (e.g., poor image quality or for certain subpopulations) (eCFR, 2025) | Frames differential error as a neutral statistical property of the dataset rather than a clinical risk of localized harm to specific patient groups (Muralidharan et al., 2024; Wu et al., 2021). | Subgroup performance evaluation indicates a statistically significant error discrepancy in dark-skinned or elderly cohorts, increasing localized risks of delayed diagnosis. |
| Missing Demographic Cohorts | A detailed summary of the current performance of the device and a summary of the performance testing conducted to support safe and effective use of the device including test methods, dataset characteristics (including demographics), testing environment, results (with confidence intervals), and a summary of sub-analyses on case distributions stratified by relevant confounders (eCFR, 2025). | Obscures structural exclusions of vulnerable populations behind broad procedural claims of overall dataset adequacy (Shick et al., 2024; Wu et al., 2021). | "Pediatric populations under 18 years were entirely excluded from validation; safety and efficacy for this demographic remain unevaluated. |
| Clinical Accountability | The device characterizes lesions based on features or information extracted from the images and provides information about the lesion(s) to the user. Diagnostic and patient management decisions are made by the clinical user (eCFR, 2025). | Transfers institutional operational and monitoring duties to clinicians without providing local drift detection protocols (Binns, 2022; Raji et al., 2020). | Post-market performance monitoring protocols are unmanaged by the sponsor; adopting health systems must perform independent local validation to detect drift. |

**References**

Alfrink, K., Keller, I., Kortuem, G., & Doorn, N. (2023). Contestable AI by Design: Towards a Framework. *Minds and Machines*, *33*(4), 613–639. https://doi.org/10.1007/s11023-022-09611-z

Ananny, M., & Crawford, K. (2018). Seeing without knowing: Limitations of the transparency ideal and its application to algorithmic accountability. *New Media & Society*, *20*(3), 973–989. https://doi.org/10.1177/1461444816676645

Binns, R. (2022). Human Judgment in algorithmic loops: Individual justice and automated decision‐making. *Regulation & Governance*, *16*(1), 197–211. https://doi.org/10.1111/rego.12358

eCFR. (2025, June 26). *21 CFR Part 892—Radiology Devices*. https://www.ecfr.gov/current/title-21/chapter-I/subchapter-H/part-892

US FDA. (2025). *Marketing Submission Recommendations for a Predetermined Change Control Plan for Artificial Intelligence-Enabled Device Software Functions: Guidance for Industry and Food and Drug Administration Staff.* https://www.fda.gov/regulatory-information/search-fda-guidance-documents/marketing-submission-recommendations-predetermined-change-control-plan-artificial-intelligence

US FDA, Health Canada, & Medicines and Healthcare products Regulatory Agency. (2021). *Good Machine Learning Practice for Medical Device Development: Guiding Principles.* https://www.fda.gov/media/153486/download

US FDA, Health Canada, & Medicines and Healthcare products Regulatory Agency. (2024). *Transparency for Machine Learning-Enabled Medical Devices: Guiding Principles*. https://www.fda.gov/media/179269/download?attachment

Friedler, S. A., Scheidegger, C., & Venkatasubramanian, S. (2016). *On the (im)possibility of fairness* (arXiv:1609.07236). arXiv. https://doi.org/10.48550/arXiv.1609.07236

Lyons, H., Velloso, E., & Miller, T. (2021). Conceptualising Contestability: Perspectives on Contesting Algorithmic Decisions. *Proceedings of the ACM on Human-Computer Interaction*, *5*(CSCW1), 1–25. https://doi.org/10.1145/3449180

Mehta, V., Komanduri, A., Bhadouriya, R. S., Mehta, V., Johnson, M. D., Shrestha, P., Nikolov, M., Jain, B., Shah, N., & Schulman, K. (2025). Evaluating transparency in AI/ML model characteristics for FDA-reviewed medical devices. *npj Digital Medicine*, *8*(1), 673. https://doi.org/10.1038/s41746-025-02052-9

Mulligan, D. K., Kluttz, D., & Kohli, N. (2018). Contestability and Professionals: From Explanations to Engagement with Algorithmic Systems. *SSRN Electronic Journal*. https://doi.org/10.2139/ssrn.3311894

Muralidharan, V., Adewale, B. A., Huang, C. J., Nta, M. T., Ademiju, P. O., Pathmarajah, P., Hang, M. K., Adesanya, O., Abdullateef, R. O., Babatunde, A. O., Ajibade, A., Onyeka, S., Cai, Z. R., Daneshjou, R., & Olatunji, T. (2024). A scoping review of reporting gaps in FDA-approved AI medical devices. *npj Digital Medicine*, *7*(1), 273. https://doi.org/10.1038/s41746-024-01270-x

Raji, I. D., Kumar, I. E., Horowitz, A., & Selbst, A. (2022). The Fallacy of AI Functionality. *2022 ACM Conference on Fairness Accountability and Transparency*, 959–972. https://doi.org/10.1145/3531146.3533158

Raji, I. D., Smart, A., White, R. N., Mitchell, M., Gebru, T., Hutchinson, B., Smith-Loud, J., Theron, D., & Barnes, P. (2020). Closing the AI accountability gap: Defining an end-to-end framework for internal algorithmic auditing. *Proceedings of the 2020 Conference on Fairness, Accountability, and Transparency*, 33–44. https://doi.org/10.1145/3351095.3372873

Selbst, A. D., Boyd, D., Friedler, S. A., Venkatasubramanian, S., & Vertesi, J. (2019). Fairness and Abstraction in Sociotechnical Systems. *Proceedings of the Conference on Fairness, Accountability, and Transparency*, 59–68. https://doi.org/10.1145/3287560.3287598

Shick, A. A., Webber, C. M., Kiarashi, N., Weinberg, J. P., Deoras, A., Petrick, N., Saha, A., & Diamond, M. C. (2024). Transparency of artificial intelligence/machine learning-enabled medical devices. *npj Digital Medicine*, *7*(1), 21. https://doi.org/10.1038/s41746-023-00992-8

Windecker, D., Baj, G., Shiri, I., Kazaj, P. M., Kaesmacher, J., Gräni, C., & Siontis, G. C. M. (2025). Generalizability of FDA-Approved AI-Enabled Medical Devices for Clinical Use. *JAMA Network Open*, *8*(4), e258052. https://doi.org/10.1001/jamanetworkopen.2025.8052

Wu, E., Wu, K., Daneshjou, R., Ouyang, D., Ho, D. E., & Zou, J. (2021). How medical AI devices are evaluated: Limitations and recommendations from an analysis of FDA approvals. *Nature Medicine*, *27*(4), 582–584. https://doi.org/10.1038/s41591-021-01312-x
